# Drug-specific risks of acute kidney injury associated with triple whammy therapy: a nationwide self-controlled case series study in Japan

**DOI:** 10.1186/s40780-026-00560-8

**Published:** 2026-03-07

**Authors:** Yuki Kunitsu, Rina Abe, Keiko Ikuta, Daiki Hira, Shunsaku Nakagawa, Masahiro Tsuda, Tomohiro Terada

**Affiliations:** 1 Department of Clinical Pharmacology and Therapeutics, Kyoto University Hospital, 54 Shogoin-Kawahara-cho, Sakyo-ku, Kyoto, 606-8507 Japan; 2 Graduate School of Pharmaceutical Sciences, Kyoto University, 46-29 Yoshida-Shimo-Adachi-cho, Sakyo-ku, Kyoto, 606-8501 Japan

**Keywords:** Acute kidney injury, Non-steroidal anti-inflammatory drugs, Renin-angiotensin system inhibitors, Diuretics, Triple whammy, Self-controlled case series

## Abstract

**Background:**

Concomitant use of renin–angiotensin system inhibitors (RASIs), diuretics, and nonsteroidal anti-inflammatory drugs (NSAIDs), commonly referred to as the “triple whammy” (TW), is known to increase the risk of acute kidney injury (AKI). However, the relative contribution of each drug component and the influence of their pharmacological subclasses remain unclear.

**Methods:**

A self-controlled case series (SCCS) study was conducted in Japan using a nationwide claims database (April 2014 to January 2024). Patients with at least one AKI episode (the International Classification of Diseases, 10th Edition code N17X) and prescriptions for RASIs, diuretics, or NSAIDs were included. The incidence rate ratios (IRRs) and 95% confidence intervals (CIs) for AKI were estimated using conditional Poisson regression to compare the exposed and unexposed periods within individuals. Sensitivity analyses accounted for the time-varying nephrotoxic exposure to antibiotics, antiviral agents, and iodinated contrast media.

**Results:**

Among the 92,880 eligible patients, 109,351 AKI events occurred during 129 million person-days of follow-up. The risk of AKI increased with the number of concomitant drug classes used. Concomitant use of two drug class combinations, that is, diuretics and NSAIDs, conferred the greatest risk, and during TW exposure, the risk was not significantly higher than that observed with diuretics and NSAIDs (IRR 1.01 [0.86–1.19]). The risk of AKI increased with both the number and type of diuretics used, with loop diuretics demonstrating the highest risk. No significant differences were found between angiotensin-converting enzyme inhibitors and angiotensin II receptor blockers, and the higher AKI risk observed with nonselective COX inhibitors compared with COX-2-selective agents disappeared after adjustment for time-dependent covariates.

**Conclusions:**

In this nationwide SCCS study, the AKI risk associated with TW therapy was primarily driven by the concomitant use of diuretics and NSAIDs, rather than RASIs. The risk varied according to the number and type of diuretics used, emphasizing the importance of cautious prescription and close renal monitoring during periods of combined use of diuretics and NSAIDs.

**Supplementary Information:**

The online version contains supplementary material available at 10.1186/s40780-026-00560-8.

## Background

Acute kidney injury (AKI) is a serious adverse event associated with the concomitant use of renin–angiotensin system inhibitors (RASIs), diuretics, and nonsteroidal anti-inflammatory drugs (NSAIDs), a combination commonly known as the “triple whammy” (TW) [1–5]. These drugs can interact pharmacologically to impair renal hemodynamics. RASIs cause efferent arteriolar dilation, diuretics reduce intravascular volume, and NSAIDs inhibit prostaglandin-mediated afferent vasodilation, drastically declining the glomerular filtration [6]. Although the TW interaction has been recognized for more than two decades, its magnitude and determinants in real-world practice remain poorly understood.

Previous pharmacoepidemiologic studies have reported an increased risk of AKI during TW exposure [4, 7, 8]; however, their definitions of concurrent use and exposure duration varied, and the contribution of each component drug has not been fully elucidated. We have previously demonstrated that 65% of TW cases were triggered by the addition of NSAIDs to ongoing RASI + diuretic therapy, and the median time to AKI onset after NSAID initiation was only 6.5 days [9, 10]. These findings suggest that the introduction of NSAIDs is a critical precipitating factor. However, whether the risk of AKI differs according to the pharmacological properties of NSAIDs, RASIs, and diuretics remains unclear. NSAIDs can be broadly categorized into nonselective cyclooxygenase inhibitors (nsCOXi) and selective cyclooxygenase-2 inhibitors (sCOX-2i). Although COX-2-selective agents, such as celecoxib, are often considered to have a lower renal risk profile, COX-2 expression in the kidneys and its role in maintaining renal perfusion indicate that sCOX2i may not be entirely benign [11–14]. Similarly, among RASIs, angiotensin-converting enzyme inhibitors (ACEIs) and angiotensin II receptor blockers (ARBs) differ in their pharmacological effects and clinical outcomes, although previous studies have reported inconsistent findings [15–19]. In addition, the type and number of diuretics used may impact renal susceptibility to hemodynamic stress [20–23]. However, no large-scale study has comprehensively quantified the drug class-specific risk for AKI in a TW setting. In addition, most previous studies have depended on cohort or case–control designs, which are susceptible to between-person confounding factors, such as underlying comorbidities and chronic kidney disease severity. The self-controlled case series (SCCS) design offers a methodological advantage by comparing the exposed and unexposed periods within the same individual, inherently controlling for time-invariant confounders [24–28]. Accordingly, we applied an SCCS approach to quantify the drug-specific risks of AKI associated with TW therapy, focusing on differences across NSAID, RASI, and diuretic classes in a nationwide Japanese claims database.

## Methods

### Data sources

Data were obtained from the DeSC database (DeSC Healthcare Inc., Tokyo, Japan), a nationwide claims database that includes anonymized medical and pharmacy claims for individuals covered by employment-based health insurance, the National Health Insurance, and the Late-Stage Elderly Health Care System. The database contains information on patient demographics, diagnostic data linked to the International Classification of Diseases, 10th Edition (ICD-10) categories, prescription records mapped to Anatomical Therapeutic Chemical (ATC) drug classes, and medical procedures. All data were fully anonymized by a database provider before being made available for research. The database contains medical claims data of approximately 12 million individuals across several Japanese insurance systems, with an age distribution representative of the Japanese population [29, 30]. 

### Study design

This study employed an SCCS design to estimate the relative incidence of AKI during exposure to a combination of RASIs, diuretics, and NSAIDs. The SCCS method inherently controls for all time-invariant confounders by comparing exposed and unexposed periods within the same individual [24–28]. This within-person design is particularly advantageous for pharmacoepidemiologic evaluations of transient exposures and acute outcomes, such as drug-induced AKI, where between-person confounding can be substantial [27, 28]. The observation window for each individual was defined as 6 months after database enrollment until censoring. Because patients are known to have an increased risk of AKI recurrence shortly after an initial episode [31–33], the 90 days following each AKI event were excluded from the risk and observation windows to minimize bias due to event dependence.

### Study population

Patients who experienced at least one episode of AKI between April 2014 and January 2024 were included if they were prescribed at least one of the following drug classes: RASIs, diuretics, or NSAIDs, excluding on-demand or topical formulations. The observation period for each patient was defined as the interval from 6 months after database enrollment until censoring. The follow-up ended at the earliest occurrence of either (1) disappearance from the database (loss of insurance eligibility) or (2) the onset of continuous dialysis. Continuous dialysis was defined as hemodialysis occurring at least once every 7 days for a period of 30 days or longer. Patients who were younger than 20 years of age at the time of database registration or had a history of continuous dialysis within 6 months of registration were excluded.

### Exposure

RASIs, diuretics, NSAIDs, and their subclasses were defined according to the ATC classification system (Supplementary Table S1). The start date of the exposure was defined as the prescription dispensing date. The duration of use of each drug was calculated based on the number of days supplied for each prescription. A 30-day grace period was allowed at the end of each exposure period to account for potential delays in medication refilling or follow-up visits. Accordingly, the risk window for each medication was defined as the entire prescribed duration from the dispensing date through the end of the supply, including the grace period, because the aim of this study was to evaluate AKI risk during ongoing medication use in real-world clinical settings rather than immediate pharmacologic effects. As-needed prescriptions were excluded because the actual timing and duration of use could not be reliably determined from claims data.

### Outcome definition

The primary outcome of the study was the new diagnosis of AKI, identified using the ICD-10 code N17X. Previous studies have reported that ICD-10 code N17X has moderate sensitivity and high specificity for identifying AKI [34]. The date of the procedure with Japanese procedure code J038 or C102 was defined as the date of dialysis.

### Statistical analysis

Incidence rate ratios (IRRs) and 95% confidence intervals (CIs) for AKI during each exposure period were estimated using conditional Poisson regression, comparing the incidence during exposure periods with that during unexposed baseline periods within each individual. In each analysis, only patients who experienced at least one AKI event during the observation window and had defined exposure and baseline periods (either or both containing AKI events) were included in the SCCS dataset. This approach ensured that comparisons were restricted to individuals who contributed person-time in both the risk and reference periods, which is consistent with the standard SCCS framework. The exposure status was modeled as a categorical time-varying variable defined according to the specific analytical objective. For the main analysis, the exposure categories represented combinations of the three study drug classes (RASI, diuretics, and NSAIDs). Exposure in subsequent subgroup analyses was further classified based on drug subclass characteristics, such as COX selectivity among NSAIDs, diuretic type, and RASI class (ACEI or ARB), to evaluate drug-specific differences in AKI risk. As individual patients could transition between different exposure states during the observation period, each individual contributed person-time to multiple categories, as applicable. The significance level was set at *p* < 0.05. The analyses were performed using R version 4.4.1 (R Foundation for Statistical Computing, Vienna, Austria).

### Sensitivity analysis

Several sensitivity analyses were performed to assess the robustness of the findings. First, the use of antibiotics, antiviral agents, and iodinated contrast media was incorporated as time-varying covariates in the SCCS models to account for time-dependent factors that might influence the risk of AKI. These agents were selected because they are known to increase AKI risk through infection-related or nephrotoxic mechanisms, potentially confounding the temporal association between drug exposure and AKI events [35–38]. Antibiotics were defined as systemic antibacterial agents classified under ATC code J01, antiviral agents under code J05, and iodinated contrast media under code V08A. Exposure periods for antibiotics and antiviral agents were defined based on the prescription date and number of treatment days recorded in the claims data. For iodinated contrast media, the 30 days after each administration were defined as the risk window for capturing delayed nephrotoxic effects. These periods were modeled as separate indicator variables in a conditional Poisson regression to adjust for their potential impact on AKI occurrence. Second, we conducted an additional analysis in which both the medication-related periods described above and hospitalization periods were simultaneously included as time-varying covariates to further address potential confounding by acute illness severity. Third, we performed stratified SCCS analyses separately for patients aged ≥ 75 years and those aged < 75 years to evaluate whether age modified the association between drug exposure and AKI risk. Finally, an analysis restricted to the period up to the first AKI event was conducted to eliminate the potential influence of recurrent AKI episodes on within-person comparisons.

### Ethics

This study was conducted in accordance with the principles of the Declaration of Helsinki. According to the Japanese Ethical Guidelines for Medical and Biological Research Involving Human Subjects, the use of fully anonymized secondary data was exempted from institutional review board approval and informed consent requirements. Therefore, informed consent and institutional review board approval were not required.

## Results

A total of 92,880 patients were included in this analysis. The median age was 79 years (range, 20–108 years), and 56.6% of the patients were male. Across the entire observation period—including person-time after AKI events—the cohort accumulated 129,055,196 person-days of follow-up (median, 1,431 days; range, 3–3,410 days). During this period, 109,351 AKI events were identified. In the analyses evaluating the association between medication exposure and AKI risk, only person-time before each AKI event was used. The distribution of the patients according to their exposure to RASIs, diuretics, and NSAIDs is shown in Supplementary Table S2.

### Main analysis

**Fig. 1:**
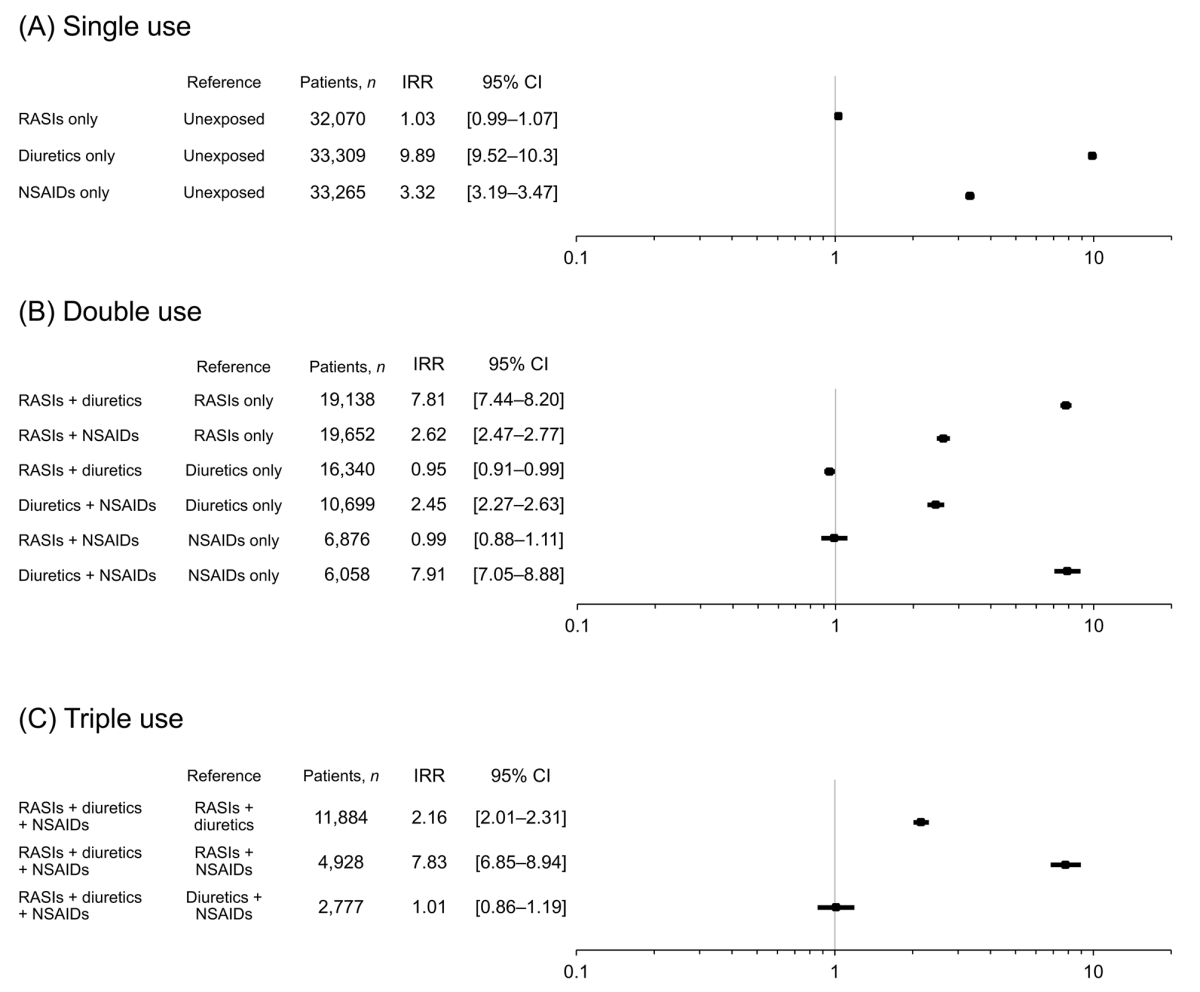
Incidence rate ratios (IRRs) and 95% confidence intervals (CIs) for acute kidney injury (AKI) associated with single, double, and triple use of renin–angiotensin system inhibitors (RASIs), diuretics, and nonsteroidal anti-inflammatory drugs (NSAIDs). IRRs were estimated using conditional Poisson regression within a self-controlled case series design. Unexposed indicates person-time during which none of the three study drug classes—RASIs, diuretics, or NSAIDs—were prescribed. “Reference” refers to the control period for each comparison

The risk of AKI increased with an increase in the number of concomitant drug classes used (Fig. 1). Compared with periods unexposed to the study drugs, the IRR [95% CI] was 1.03 [0.99–1.07] for RASI use, 9.89 [9.52–10.3] for diuretic use, and 3.32 [3.19–3.47] for NSAID use. When the two drug classes were used concomitantly, no significant increase in AKI risk was observed for the RASI and diuretic combination compared with diuretic use alone, and the RASI and NSAID combination did not show a significant increase in risk compared with NSAID use alone. The risk of AKI was not significantly different from that observed during periods of concomitant diuretic and NSAID use (IRR 1.01 [0.86–1.19]) during periods of triple-drug use (TW).

Detailed analyses of diuretic use are summarized in Table 1. The IRR increased with the number of diuretic agents prescribed: 6.57 [5.70–7.59] for one diuretic compared with no diuretics, 6.52 [5.34–7.97] for two diuretics compared with one, and 3.68 [2.45–5.51] for three or more diuretics compared with two. Among diuretic subclasses, loop diuretics were associated with the highest risk (IRR 13.6 [11.4–16.2]).

**Table 1:** Incidence rate ratios for AKI according to the number and type of concomitant diuretics under RASI and NSAID exposure

Diuretic category	Reference	Patients, *n*	IRR	[95% CI]
Exactly 1 diuretic				
Any diuretics	No diuretics (RASIs + NSAIDs only)	4,430	6.57	[5.70 − 7.59]
Loop diuretics	No diuretics (RASIs + NSAIDs only)	3,025	13.6	[11.4 − 16.2]
Potassium-sparing diuretics	No diuretics (RASIs + NSAIDs only)	513	2.69	[1.79 − 4.06]
Thiazide diuretics	No diuretics (RASIs + NSAIDs only)	989	1.38	[1.01 − 1.89]
Thiazide-like diuretics	No diuretics (RASIs + NSAIDs only)	305	2.06	[1.22 − 3.50]
V2RA	No diuretics (RASIs + NSAIDs only)	20	8.12	[0.97 − 67.7]
Exactly 2 diuretics				
Any combination	1 diuretic (any diuretics)	2,200	6.52	[5.34 − 7.97]
Loop + potassium-sparing	Loop only	968	5.77	[4.20 − 7.92]
Loop + thiazide	Loop only	255	2.61	[1.44 − 4.74]
Loop + thiazide-like	Loop only	61	6.47	[2.16 − 19.4]
Loop + V2RA	Loop only	235	2.81	[1.46 − 5.41]
Loop + potassium-sparing	Potassium-sparing only	295	4.34	[2.39 − 7.87]
Potassium-sparing + thiazide	Potassium-sparing only	42	0.49	[0.12 − 1.99]
Potassium-sparing + thiazide-like	Potassium-sparing only	14	2.08	[0.28 − 15.6]
Potassium-sparing + V2RA	Potassium-sparing only	9	NE	
Loop + thiazide	Thiazide only	391	21.4	[12.7 − 36.0]
Potassium-sparing + thiazide	Thiazide only	97	12.3	[3.24 − 46.5]
Thiazide + thiazide-like	Thiazide only	31	NE	
Thiazide + V2RA	Thiazide only	5	NE	
Loop + thiazide-like	Thiazide-like only	84	116	[27.1 − 498]
Potassium-sparing + thiazide-like	Thiazide-like only	18	NE	
Thiazide + thiazide-like	Thiazide-like only	32	NE	
Thiazide-like + V2RA	Thiazide-like only	2	NE	
Loop + V2RA	V2RA only	15	7.13	[0.38 − 132]
Potassium-sparing + V2RA	V2RA only	8	NE	
Thiazide + V2RA	V2RA only	2	NE	
Thiazide-like + V2RA	V2RA only	0	NE	
≥ 3 diuretics	V2RA only			
Any combination	2 diuretics (any combination)	550	3.68	[2.45 − 5.51]

Any diuretic indicates the use of exactly one diuretic agent from any class (loop, potassium-sparing, thiazide, thiazide-like, or V2RA). Any combination indicates the use of two or three diuretics from any class. The reference refers to the designated control exposure period for each comparison. NE (not estimable) indicates that the IRR could not be calculated because of insufficient AKI events

Abbreviations: AKI, acute kidney injury. CI, confidence interval. IRR: incidence rate ratio. NSAIDs, nonsteroidal anti-inflammatory drugs. RASIs, renin–angiotensin system inhibitors. V2RA: vasopressin V2 receptor antagonist

As shown in Table 2, no significant difference in the risk of AKI was observed between the ACEI and ARB groups during the TW period. In contrast, among NSAIDs, nsCOXi were associated with a higher AKI risk than sCOX-2i (IRR 1.35 [1.09–1.67]) (Table 3).

**Table 2:** Incidence rate ratios for AKI according to the type of concomitant RASIs under diuretic and NSAID exposure

Exposure category	Reference	Patients, *n*	IRR	[95% CI]
ARB + diuretics + NSAIDs	ACEI + diuretics + NSAIDs	308	1.45	[0.85 − 2.48]
ARB + exactly 1 diuretic + NSAIDs	ACEI + 1 diuretic + NSAIDs	160	1.04	[0.44 − 2.42]

The reference refers to the designated control exposure period for each comparison

ACEI, angiotensin-converting enzyme inhibitor. AKI, acute kidney injury. ARB, angiotensin II receptor blocker. CI, confidence interval. IRR: incidence rate ratio. NSAIDs, nonsteroidal anti-inflammatory drugs. RASIs, renin–angiotensin system inhibitors

**Table 3:** Incidence rate ratios for AKI according to the type of concomitant NSAIDs under RASI and diuretic exposure

Exposure category	Reference	Patients, *n*	IRR	[95% CI]
RASIs + diuretics + nsCOXi	RASIs + diuretics + sCOX-2i	2,277	1.35	[1.09 − 1.67]
RASIs + exactly 1 diuretic + nsCOXi	RASIs + exactly 1 diuretic + sCOX-2i	1,616	1.59	[1.20 − 2.09]

The reference refers to the designated control exposure period for each comparison

Abbreviations: AKI, acute kidney injury. CI, confidence interval. IRR: Incidence rate ratio. NSAIDs, nonsteroidal anti-inflammatory drugs. nsCOXi, nonselective cyclooxygenase inhibitors. RASIs, renin–angiotensin system inhibitors. sCOX-2i: selective cyclooxygenase-2 inhibitors

### Sensitivity analyses

Sensitivity analyses incorporating the use of antibiotics, antiviral agents, and iodinated contrast media as time-varying covariates yielded results consistent with those of the main analysis (Supplementary Tables S3–S6). After adjusting for these time-dependent factors, the overall risk patterns remained stable. The adjusted IRR of AKI remained significantly higher than that during RASI and diuretic use alone during the TW period; however, it was not significantly different from that during concomitant diuretic and NSAID use. Similarly, no significant changes were observed in the relative risks of the two drug combinations after adjustment. In analyses stratified by drug class, the association between the number of diuretic agents and AKI risk persisted, and loop diuretics continued to demonstrate the highest risk among the subclasses. No material differences were found between ACEIs and ARBs. However, the previously observed higher AKI risk with nsCOXi compared with sCOX-2 inhibitors was no longer statistically significant after adjusting for time-dependent covariates. These results indicated that the main findings were robust and that the apparent difference in AKI risk between COX inhibitor types may have been partly confounded by concurrent infection- or procedure-related nephrotoxic exposures. Then when hospitalization periods were additionally incorporated as a time-varying covariate, the results remained broadly unchanged, with no meaningful shifts in the estimated IRRs across drug combinations. Age-stratified SCCS analyses (≥ 75 vs. <75 years) also demonstrated risk patterns consistent with the primary analysis, without evidence of substantial effect modification by age. In contrast, the analysis restricted to the period up to the first AKI event showed some differences from the main analysis. The IRRs associated with diuretic use were higher than in the primary analysis, and the IRR during combined RASI, diuretic, and NSAID use was significantly lower than that during diuretics plus NSAIDs alone (IRR, 0.41 [0.33–0.52]). This indicates that the relative risk comparison between these exposure states differs more clearly when the analysis is limited to first events. Overall, the findings from all sensitivity analyses support the robustness of the main results.

## Discussion

This nationwide, self-controlled case series demonstrated that the risk of AKI increased with the number of concomitant drug classes used, including RASIs, diuretics, and NSAIDs. However, the AKI risk during triple-drug exposure (TW) was not significantly different from that observed during double-drug exposure involving diuretics and NSAIDs, highlighting that the double-drug combination of diuretics and NSAIDs confers the greatest renal risk and represents the core driver of TW-associated AKI, rather than the addition of RASIs. This finding supports the hypothesis that the inhibition of prostaglandin-mediated afferent vasodilation by NSAIDs and reduced renal perfusion from diuretic-induced volume depletion constitute the core pathophysiological mechanisms underlying TW-associated AKI.

In addition, we demonstrated that AKI risk differed according to the pharmacological characteristics of the component drugs. Among diuretics, loop diuretics were associated with the highest AKI risk, consistent with their potent natriuretic effect and ability to induce marked intravascular volume depletion by inhibiting the Na⁺-K⁺-2Cl⁻ cotransporter in the loop of Henle [20]. The number of concomitant diuretic agents was positively correlated with AKI risk, suggesting an additive hemodynamic burden on the kidneys. In contrast, no material differences were observed between ACEIs and ARBs, supporting previous evidence that their impact on renal hemodynamics is broadly comparable in clinical settings [18, 19].

The main analysis revealed that nsCOXis were associated with a higher AKI risk than sCOX-2i. However, this difference was no longer statistically significant after adjusting for time-dependent covariates such as antibiotic use, antiviral therapy, and exposure to iodinated contrast media. Infection [39, 40], exposure to nephrotoxic agents such as antibiotics [35, 41], antiviral agents [36, 41], and iodinated contrast media [37, 38, 41], and related clinical conditions are well-known risk factors for AKI. In addition, NSAIDs are often prescribed for the management of fever or pain associated with infections, which may introduce treatment-selection bias in real-world data. We have previously investigated the AKI risk among patients receiving RASIs and diuretics and found no substantial difference between nsCOXi and sCOX-2i (adjusted odds ratio 0.99 [95% CI: 0.66–1.50]) [8]. Altogether, these findings suggest that COX selectivity itself does not exert a strong influence on nephrotoxicity and that both classes of NSAIDs require equal cautious use and monitoring.

In interpreting the large IRRs observed in this SCCS analysis, it is important to note that these estimates do not necessarily represent the intrinsic nephrotoxicity of the medications. Rather, they may reflect within-person contrasts between periods of higher and lower underlying clinical risk. In routine practice, diuretics are often initiated or intensified during episodes of acute clinical deterioration—such as worsening heart failure, hemodynamic instability, or volume imbalance—which themselves may increase the likelihood of AKI. Accordingly, part of the elevated IRRs for diuretics may capture these time-dependent clinical conditions in addition to any pharmacologic effects.

The absence of a clearly elevated IRR associated with RASI exposure should also be interpreted with caution. Clinical states that require diuretic use often carry substantial background risk for AKI, and such risk may overshadow smaller incremental effects attributable to RASIs within the SCCS framework. Thus, the possibility remains that modest RASI-associated risks were not readily detectable in this analysis, rather than indicating a complete absence of effect.

A sensitivity analysis restricted to the period up to the first AKI event yielded several differences from the main analysis. In particular, the IRR associated with diuretic use was higher, and the IRR during combined RASI, diuretic, and NSAID exposure was significantly lower than that during diuretics and NSAIDs alone. Such discrepancies may arise because the clinical context surrounding an initial AKI episode often differs from that of subsequent episodes. For example, treatment intensity, prescribing patterns, and patient susceptibility may change after an event, altering the within-person contrasts that underpin SCCS estimation. These findings suggest that exposure–event associations may vary depending on whether recurrent events are included, although the primary conclusions of this study remained robust across analyses.

Previous studies have predominantly focused on the high risk of AKI associated with the three-drug combination of RASIs, diuretics, and NSAIDs (TW) [1–5]. It is plausible that RASIs contribute to the development of TW-associated AKI through efferent arteriolar dilation and reduced glomerular filtration pressure, as suggested in previous pathophysiologic models and clinical observations [1, 4, 5]. However, our findings refine this understanding by highlighting that the combination of diuretics and NSAIDs appears to play a predominant role in precipitating AKI, regardless of the presence of RASIs. This does not contradict the established concept that RASIs can interact with other nephrotoxic factors but rather suggests that their contribution is smaller in magnitude than the hemodynamic impact of volume depletion and prostaglandin inhibition. Moreover, RASIs may exert long-term renal-protective effects [42, 43], which could partly offset their short-term hemodynamic effects in certain contexts. Altogether, these findings suggest that although RASIs remain mechanistically involved in the TW pathway, the diuretic–NSAID combination represents the principal driver of AKI risk in real-world settings. When diuretics and NSAIDs are used concomitantly, renal risk should be carefully evaluated, and appropriate monitoring should be implemented.

This study had several limitations. First, AKI was identified using diagnostic codes, which may have limited sensitivity despite their high specificity [34]. Furthermore, recent validation research from Japan [44] has demonstrated that ICD-10–based definitions of AKI in claims data may have a limited positive predictive value. Although such limitations are inherent to administrative databases and do not invalidate the within-person comparisons used in this study, further efforts to refine and validate claims-based AKI definitions in Japan are warranted to improve the accuracy of future pharmacoepidemiologic research. Second, prescription records do not guarantee actual drug ingestion, and the use of over-the-counter NSAIDs cannot be captured. Moreover, although dose information is recorded in the claims data, meaningful dose–response evaluation was not feasible because diuretics encompass multiple subclasses without standardized dose conversion, are frequently used in combination, and prescribed doses do not necessarily reflect the actual amount taken. Third, because individuals cannot be continuously tracked across insurance transitions in this database, changes in insurance status—particularly the transition to the late-stage elderly healthcare system—may result in artificial censoring or duplicate entries. To minimize misclassification, we implemented a 6-month look-back period after database entry and excluded time immediately following insurance transitions where medication history could not be reliably confirmed. Nonetheless, some degree of uncertainty may remain, and this should be considered when interpreting the findings. Fourth, unmeasured and transient factors such as hydration status or acute illness could not be directly incorporated, which may have introduced residual confounding factors. Fifth, the generalizability of the findings is limited to populations with similar prescription patterns and healthcare access, such as in Japan. In addition, because the study population was predominantly elderly, the results may not be fully generalizable to younger individuals, who were underrepresented in this dataset. Although the age-stratified sensitivity analysis demonstrated that the overall risk patterns were generally consistent between patients aged < 75 and ≥ 75 years, the generalizability of our findings to younger populations should be interpreted with caution. The present cohort is predominantly composed of elderly individuals, who typically have distinct clinical trajectories, comorbidity burdens, and prescribing patterns compared with younger adults. In addition, younger individuals were substantially underrepresented in this claims database, resulting in a limited number of AKI events available for evaluation in that subgroup. Therefore, while the sensitivity analysis suggests that the observed associations are not exclusively driven by advanced age, further research in cohorts with a broader age distribution will be necessary to confirm whether these findings are applicable to younger populations.

Nevertheless, the large sample size, nationwide coverage, and self-controlled design strengthen the validity of the findings.

## Conclusions

We demonstrate that the risk of AKI associated with TW therapy is primarily driven by the concomitant use of diuretics and NSAIDs rather than RASIs. The risk of AKI varied according to both the number and type of diuretics used, with loop diuretics conferring the greatest risk. These results highlight the significance of monitoring renal function during combined diuretic and NSAID use, and underscore the need for caution when co-prescribing nephrotoxic agents in clinical practice.

## Supplementary Information

Below is the link to the electronic supplementary material.

Supplementary Material 1

## Data Availability

The data that support the findings of this study are available from DeSC Healthcare Inc. However, restrictions apply to the availability of these data, which were used under the license for the current study and are not publicly available. The data are available from the authors upon reasonable request and with permission from DeSC Healthcare Inc.

## Abbreviations

ACEIs: Angiotensin-converting enzyme inhibitors
AKI: Acute kidney injury
ARBs: Angiotensin II receptor blockers
ATC: Anatomical Therapeutic Chemical
CI: Confidence interval
ICD-10: International Classification of Diseases:10th Edition
IRR: Incidence rate ratio
NSAIDs: Non-steroidal anti-inflammatory drugs
nsCOXi: Nonselective cyclooxygenase inhibitors
RASIs: Renin-angiotensin system inhibitors
SCCS: Self-controlled case series
sCOX-2i: Selective cyclooxygenase-2 inhibitors
TW: Triple Whammy
V2RA: Vasopressin V2 receptor antagonist
