# Supplementary material for: Drug-specific risks of acute kidney injury associated with triple whammy therapy: a nationwide self-controlled case series study in Japan
Source: J Pharm Health Care Sci. 2026 Mar 7;12:42. doi: 10.1186/s40780-026-00560-8 (PMC13081389; doi:10.1186/s40780-026-00560-8)
Supplement: Supplementary file 1 — Supplementary Material 1 [file 40780_2026_560_MOESM1_ESM.pdf]

Supplementary Materials

Drug-Specific Risks of Acute Kidney Injury Associated with Triple Whammy Therapy: A Nationwide Self-Controlled Case Series Study in Japan

Authors: Yuki Kunitsu, Rina Abe, Keiko Ikuta, Daiki Hira, Shunsaku Nakagawa, Masahiro Tsuda, and Tomohiro Terada

Corresponding author: Yuki Kunitsu
Department of Clinical Pharmacology and Therapeutics, Kyoto University Hospital, 54 Shogoin-Kawahara-cho, Sakyo-ku, Kyoto, 606-8507, Japan
Email: ykunitsu@kuhp.kyoto-u.ac.jp

Supplementary Table S1 Definition of RASIs, diuretics, and NSAIDs
Supplementary Table S2 Distribution of patients according to their exposure status to RASIs, diuretics, and NSAIDs
Supplementary Table S3 Incidence rate ratios for AKI according to single-, double-, and triple-drug exposure categories in sensitivity analyses
Supplementary Table S4 Incidence rate ratios for AKI according to the number and type of concomitant diuretics under RASI and NSAID exposure in the sensitivity analyses
Supplementary Table S5 Incidence rate ratios for AKI according to the type of concomitant RASIs under diuretic and NSAID exposure in sensitivity analyses
Supplementary Table S6 Incidence rate ratios for AKI according to the type of concomitant NSAIDs under RASI and diuretic exposure in the sensitivity analyses

Supplementary Table S1 Definition of RASIs, diuretics, and NSAIDs

Category	Drug name
RASIs	
ACEIs	Alacepril, benazepril hydrochloride, captopril, cilazapril hydrate, delapril hydrochloride, enalapril maleate, imidapril hydrochloride, lisinopril hydrate, perindopril erbumin, quinapril hydrochloride, temocapril hydrochloride, and trandolapril
ARBs	Azilsartan, candesartan cilexetil, irbesartan, losartan potassium, olmesartan medoxomil, sacubitoril valsartan sodium hydrate, telmisartan, and valsartan
Direct renin inhibitor	Aliskiren fumarate
Diuretics	
Loop diuretics	Azosemide, bumetanide, furosemide, and trasemide
Potassium-sparing diuretics	Eplerenone, esaxelenone, potassium kanrenoate, spironolactone, and triamterene
Thiazide diuretics	Benzylhydrochlorothiazide, hydrochlorothiazide, and trichloromethiazide
Thiazide-like diuretics	Indapamide, meflucide, methycran, and tripamide
Vasopressin V2 receptor antagonists	Tolvaptan
NSAIDs	
nsCOX-i	Acemetacin, aluminium flufenamate, ampiroxicam, diclofenac sodium, flurbiprofen, flurbiprofen axetil, ibuprofen, indomethacin, indomethacin farnesyl, ketoprofen, loxoprofen sodium hydrate, mefenamic acid, moffezolac, naproxen, oxaprozin, piroxicam, planoprofen, proglumetacin maleate, surindac, and thiaprofenic acid
sCOX2-i	Celecoxib, etodolac, lornoxicam, meloxicam, nabumetone, and zartoprofen

Abbreviations: ACEIs, angiotensin-converting enzyme inhibitors. ARBs, angiotensin receptor blockers. NSAIDs, non-steroidal anti-inflammatory drugs. nsCOX-i, nonselective cyclooxygenase inhibitors. RASIs, renin-angiotensin system inhibitors. sCOX2-i, selective cyclooxygenase-2 inhibitors.

Supplementary Table S2 Distribution of patients according to their exposure status to RASIs, diuretics, and NSAIDs

RASIs	Diuretics	NSAIDs	Target patients, *n*	AKI, *n*	Duration, days
Overall observation period					
-	-	-	82,507	36,290	58,301,497
+	-	-	43,208	18,339	29,628,193
-	+	-	49,379	21,039	13,892,363
-	-	+	36,966	5,507	4,275,816
+	+	-	36,425	19,396	16,828,890
+	-	+	22,107	3,154	3,201,760
-	+	+	15,506	2,749	1,165,418
+	+	+	14,946	2,877	1,761,259
Analysis period excluding post-AKI					
-	-	-	74,654	32,003	54,776,784
+	-	-	41,969	16,597	28,551,628
-	+	-	39,205	18,127	12,625,643
-	-	+	34,163	4,884	4,050,805
+	+	-	32,831	17,308	15,954,898
+	-	+	21,111	2,892	3,094,674
-	+	+	12,668	2,419	1,070,413
+	+	+	13,530	2,577	1,684,244

AKI, acute kidney injury. NSAIDs, non-steroidal anti-inflammatory drugs. RASIs and RAS inhibitors.

Supplementary Table S3 Incidence rate ratios for AKI according to single-, double-, and triple-drug exposure categories in sensitivity analyses

Exposure category	Reference	Patients, n	IRR	[95% CI]
Sensitivity analysis adjusting for exposure to antibiotics, antivirals, and iodinated contrast media				
Single use				
- RASIs only	Unexposed	32,070	1.03	[0.996−1.07]
- Diuretics only	Unexposed	33,309	4.88	[4.71−5.06]
- NSAIDs only	Unexposed	33,265	1.42	[1.37−1.48]
Double use				
- RASIs + diuretics	RASIs only	19,138	5.60	[5.34−5.87]
- RASIs + NSAIDs	RASIs only	19,652	1.34	[1.27−1.42]
- RASIs + diuretics	Diuretics only	16,340	1.13	[1.08−1.18]
- Diuretics + NSAIDs	Diuretics only	10,699	1.53	[1.42−1.64]
- RASIs + NSAIDs	NSAIDs only	6,876	1.13	[1.01−1.27]
- Diuretics + NSAIDs	NSAIDs only	6,058	5.79	[5.17−6.47]
Triple use				
- RASIs + diuretics + NSAIDs	RASIs + diuretics	11,884	1.28	[1.20−1.37]
- RASIs + diuretics + NSAIDs	RASIs + NSAIDs	4,928	5.82	[5.11−6.62]
- RASIs + diuretics + NSAIDs	Diuretics + NSAIDs	2,777	1.12	[0.95−1.31]
Sensitivity analysis adjusting for antibiotics, antivirals, iodinated contrast media, and hospitalization periods				
Single use				
- RASIs only	Unexposed	32,070	1.03	[0.99−1.06]
- Diuretics only	Unexposed	33,309	5.36	[5.17−5.56]
- NSAIDs only	Unexposed	33,265	1.45	[1.39−1.51]
Double use				
- RASIs + diuretics	RASIs only	19,138	6.04	[5.76−6.33]
- RASIs + NSAIDs	RASIs only	19,652	1.34	[1.27−1.42]
- RASIs + diuretics	Diuretics only	16,340	1.12	[1.07−1.18]
- Diuretics + NSAIDs	Diuretics only	10,699	1.54	[1.43−1.65]
- RASIs + NSAIDs	NSAIDs only	6,876	1.12	[0.997−1.26]
- Diuretics + NSAIDs	NSAIDs only	6,058	6.18	[5.52−6.91]
Triple use				
- RASIs + diuretics + NSAIDs	RASIs + diuretics	11,884	1.28	[1.19−1.36]
- RASIs + diuretics + NSAIDs	RASIs + NSAIDs	4,928	6.13	[5.39−6.98]
- RASIs + diuretics + NSAIDs	Diuretics + NSAIDs	2,777	1.11	[0.94−1.30]

Sensitivity analysis stratified by age (<75 years)				
Single use				
- RASIs only	Unexposed	7,332	1.38	[1.29−1.48]
- Diuretics only	Unexposed	6,257	22.0	[20.3−23.9]
- NSAIDs only	Unexposed	9,708	4.97	[4.62−5.33]
Double use				
- RASIs + diuretics	RASIs only	3,507	6.80	[6.09−7.58]
- RASIs + NSAIDs	RASIs only	4,503	3.16	[2.82−3.54]
- RASIs + diuretics	Diuretics only	2,422	0.82	[0.73−0.93]
- Diuretics + NSAIDs	Diuretics only	1,767	2.66	[2.22−3.18]
- RASIs + NSAIDs	NSAIDs only	1,768	1.03	[0.83−1.29]
- Diuretics + NSAIDs	NSAIDs only	1,428	7.88	[6.39−9.73]
Triple use				
- RASIs + diuretics + NSAIDs	RASIs + diuretics	1,880	2.46	[2.07−2.92]
- RASIs + diuretics + NSAIDs	RASIs + NSAIDs	873	5.90	[4.42−7.89]
- RASIs + diuretics + NSAIDs	Diuretics + NSAIDs	374	0.81	[0.52−1.28]
Sensitivity analysis stratified by age (≥75 years)				
Single use				
- RASIs only	Unexposed	24,738	0.93	[0.90−0.97]
- Diuretics only	Unexposed	27,052	8.07	[7.74−8.41]
- NSAIDs only	Unexposed	23,557	2.74	[2.60−2.88]
Double use				
- RASIs + diuretics	RASIs only	15,631	8.12	[7.69−8.57]
- RASIs + NSAIDs	RASIs only	15,149	2.49	[2.33−2.66]
- RASIs + diuretics	Diuretics only	13,918	0.97	[0.92−1.02]
- Diuretics + NSAIDs	Diuretics only	8,932	2.39	[2.21−2.60]
- RASIs + NSAIDs	NSAIDs only	5,108	0.98	[0.86−1.13]
- Diuretics + NSAIDs	NSAIDs only	4,630	8.20	[7.14−9.42]
Triple use				
- RASIs + diuretics + NSAIDs	RASIs + diuretics	10,004	2.11	[1.96−2.27]
- RASIs + diuretics + NSAIDs	RASIs + NSAIDs	4,055	8.52	[7.33−9.90]
- RASIs + diuretics + NSAIDs	Diuretics + NSAIDs	2,403	1.05	[0.88−1.25]
Sensitivity analysis restricted to the period prior to the first AKI event				
Single use				
- RASIs only	Unexposed	20,044	0.54	[0.52−0.57]
- Diuretics only	Unexposed	19,595	54.4	[51.8−57.2]

- NSAIDs only	Unexposed	26,331	3.40	[3.24−3.56]
Double use				
- RASIs + diuretics	RASIs only	14,243	28.5	[26.7−30.4]
- RASIs + NSAIDs	RASIs only	16,555	2.68	[2.51−2.86]
- RASIs + diuretics	Diuretics only	8,902	0.25	[0.24−0.27]
- Diuretics + NSAIDs	Diuretics only	6,907	1.91	[1.75−2.08]
- RASIs + NSAIDs	NSAIDs only	4,402	0.75	[0.65−0.87]
- Diuretics + NSAIDs	NSAIDs only	3,992	23.2	[19.9−27.0]
Triple use				
- RASIs + diuretics + NSAIDs	RASIs + diuretics	9,587	1.84	[1.70−1.99]
- RASIs + diuretics + NSAIDs	RASIs + NSAIDs	3,692	18.4	[15.5−21.9]
- RASIs + diuretics + NSAIDs	Diuretics + NSAIDs	1,572	0.41	[0.33−0.52]

IRRs were estimated using a conditional Poisson regression in the SCCS sensitivity model, including time-dependent covariates (antibiotics, antiviral agents, and iodinated contrast media). Any diuretic indicates the use of exactly one diuretic agent from any class (loop, potassium-sparing, thiazide, thiazide-like, or V2RA). Any combination indicates the use of two or three diuretics from any class. Unexposed indicates the person-time during which none of the three study drug classes (RASIs, diuretics, or NSAIDs) were prescribed. The reference refers to the designated control exposure period for each comparison. NE (not estimable) indicates that the IRR could not be calculated because of insufficient AKI events.

Abbreviations: AKI, acute kidney injury. CI, confidence interval. IRR, incidence rate ratio. NSAIDs, nonsteroidal anti-inflammatory drugs. RASIs, renin–angiotensin system inhibitors. V2RA, vasopressin V2 receptor antagonist

Supplementary Table S4 Incidence rate ratios for AKI according to the number and type of concomitant diuretics under RASIs and NSAIDs exposure in the sensitivity analyses

Diuretic category	Reference	Patients, n	IRR	[95% CI]
Sensitivity analysis adjusting for exposure to antibiotics, antivirals, and iodinated contrast media				
Exactly 1 diuretic				
- Any diuretics	No diuretics (RASIs + NSAIDs only)	4,430	5.05	[4.40−5.81]
- Loop diuretics	No diuretics (RASIs + NSAIDs only)	3,025	8.68	[7.33−10.3]
- Potassium-sparing diuretics	No diuretics (RASIs + NSAIDs only)	513	2.55	[1.70−3.83]
- Thiazide diuretics	No diuretics (RASIs + NSAIDs only)	989	1.33	[0.97−1.81]
- Thiazide-like diuretics	No diuretics (RASIs + NSAIDs only)	305	2.30	[1.36−3.89]
- V2RA	No diuretics (RASIs + NSAIDs only)	20	9.17	[1.07−78.8]
Exactly 2 diuretics				
- Any combination	1 diuretic (any diuretics)	2,200	5.11	[4.20−6.22]
- Loop + Potassium-sparing	Loop only	968	4.89	[3.58−6.68]
- Loop + Thiazide	Loop only	255	2.71	[1.49−4.92]
- Loop + Thiazide-like	Loop only	61	6.60	[2.22−19.7]
- Loop + V2RA	Loop only	235	2.13	[1.13−4.01]
- Loop + Potassium-sparing	Potassium-sparing only	295	3.47	[1.95−6.18]
- Potassium-sparing + Thiazide	Potassium-sparing only	42	0.62	[0.16−2.38]
- Potassium-sparing + Thiazide-like	Potassium-sparing only	14	1.28	[0.17−9.80]
- Potassium-sparing + V2RA	Potassium-sparing only	9	NE	
- Loop + Thiazide	Thiazide only	391	15.0	[8.98−25.2]
- Potassium-sparing + Thiazide	Thiazide only	97	8.43	[2.16−33.0]
- Thiazide + Thiazide-like	Thiazide only	31	NE	
- Thiazide + V2RA	Thiazide only	5	NE	
- Loop + Thiazide-like	Thiazide-like only	84	70.3	[15.8−313]
- Potassium-sparing + Thiazide-like	Thiazide-like only	18	NE	
- Thiazide + Thiazide-like	Thiazide-like only	32	NE	

- Thiazide-like + V2RA	Thiazide-like only	2	NE	
- Loop + V2RA	V2RA only	15	3.69	[0.22−61.3]
- Potassium-sparing + V2RA	V2RA only	8	NE	
- Thiazide + V2RA	V2RA only	2	NE	
- Thiazide-like + V2RA	V2RA only	0	NE	
≥3 diuretics	V2RA only			
- Any combination	2 diuretics (Any combination)	550	3.24	[2.20−4.79]
Sensitivity analysis adjusting for antibiotics, antivirals, iodinated contrast media, and hospitalization periods				
Exactly 1 diuretic				
- Any diuretics	No diuretics (RASIs + NSAIDs only)	4,430	5.28	[4.59−6.07]
- Loop diuretics	No diuretics (RASIs + NSAIDs only)	3,025	9.30	[7.84−11.0]
- Potassium-sparing diuretics	No diuretics (RASIs + NSAIDs only)	513	2.53	[1.69−3.81]
- Thiazide diuretics	No diuretics (RASIs + NSAIDs only)	989	1.33	[0.97−1.82]
- Thiazide-like diuretics	No diuretics (RASIs + NSAIDs only)	305	2.39	[1.41−4.05]
- V2RA	No diuretics (RASIs + NSAIDs only)	20	9.95	[1.14−87.0]
Exactly 2 diuretics				
- Any combination	1 diuretic (any diuretics)	2,200	5.39	[4.42−6.57]
- Loop + Potassium-sparing	Loop only	968	5.15	[3.76−7.05]
- Loop + Thiazide	Loop only	255	2.74	[1.50−4.99]
- Loop + Thiazide-like	Loop only	61	6.79	[2.27−20.3]
- Loop + V2RA	Loop only	235	2.40	[1.26−4.57]
- Loop + Potassium-sparing	Potassium-sparing only	295	3.56	[1.99−6.36]
- Potassium-sparing + Thiazide	Potassium-sparing only	42	0.62	[0.16−2.39]
- Potassium-sparing + Thiazide-like	Potassium-sparing only	14	1.27	[0.16−9.82]
- Potassium-sparing + V2RA	Potassium-sparing only	9	NE	
- Loop + Thiazide	Thiazide only	391	16.2	[9.63−27.1]
- Potassium-sparing + Thiazide	Thiazide only	97	8.32	[2.13−32.4]
- Thiazide + Thiazide-like	Thiazide only	31	NE	

- Thiazide + V2RA	Thiazide only	5	NE	
- Loop + Thiazide-like	Thiazide-like only	84	80.1	[17.9−358]
- Potassium-sparing + Thiazide-like	Thiazide-like only	18	NE	
- Thiazide + Thiazide-like	Thiazide-like only	32	NE	
- Thiazide-like + V2RA	Thiazide-like only	2	NE	
- Loop + V2RA	V2RA only	15	3.67	[0.20−66.5]
- Potassium-sparing + V2RA	V2RA only	8	NE	
- Thiazide + V2RA	V2RA only	2	NE	
- Thiazide-like + V2RA	V2RA only	0	NE	
≥3 diuretics	V2RA only			
- Any combination	2 diuretics (Any combination)	550	3.58	[2.41−5.33]
Sensitivity analysis stratified by age (<75 years)				
Exactly 1 diuretic				
- Any diuretics	No diuretics (RASIs + NSAIDs only)	782	5.14	[3.76−7.02]
- Loop diuretics	No diuretics (RASIs + NSAIDs only)	475	11.5	[7.68−17.1]
- Potassium-sparing diuretics	No diuretics (RASIs + NSAIDs only)	87	1.84	[0.67−5.05]
- Thiazide diuretics	No diuretics (RASIs + NSAIDs only)	214	1.55	[0.84−2.86]
- Thiazide-like diuretics	No diuretics (RASIs + NSAIDs only)	66	2.37	[0.94−5.98]
- V2RA	No diuretics (RASIs + NSAIDs only)	3	NE	
Exactly 2 diuretics				
- Any combination	1 diuretic (any diuretics)	267	5.78	[3.18−10.5]
- Loop + Potassium-sparing	Loop only	112	6.7	[2.27−19.5]
- Loop + Thiazide	Loop only	23	13.1	[1.01−169]
- Loop + Thiazide-like	Loop only	4	5.62	[0.30−106]
- Loop + V2RA	Loop only	32	0.75	[0.06−9.68]
- Loop + Potassium-sparing	Potassium-sparing only	43	2.30	[0.57−9.35]
- Potassium-sparing + Thiazide	Potassium-sparing only	7	NE	
- Potassium-sparing + Thiazide-like	Potassium-sparing only	2	NE	

- Potassium-sparing + V2RA	Potassium-sparing only	3	NE	
- Loop + Thiazide	Thiazide only	36	11.0	[2.46−48.7]
- Potassium-sparing + Thiazide	Thiazide only	14	NE	
- Thiazide + Thiazide-like	Thiazide only	1	NE	
- Thiazide + V2RA	Thiazide only	0	NE	
- Loop + Thiazide-like	Thiazide-like only	6	62.7	[4.28−919]
- Potassium-sparing + Thiazide-like	Thiazide-like only	5	NE	
- Thiazide + Thiazide-like	Thiazide-like only	3	NE	
- Thiazide-like + V2RA	Thiazide-like only	0	NE	
- Loop + V2RA	V2RA only	2	NE	
- Potassium-sparing + V2RA	V2RA only	1	NE	
- Thiazide + V2RA	V2RA only	0	NE	
- Thiazide-like + V2RA	V2RA only	0	NE	
≥3 diuretics	V2RA only			
- Any combination	2 diuretics (Any combination)	63	3.77	[1.13−12.6]
Sensitivity analysis stratified by age (≥75 years)				
Exactly 1 diuretic				
- Any diuretics	No diuretics (RASIs + NSAIDs only)	3,648	7.14	[6.07−8.39]
- Loop diuretics	No diuretics (RASIs + NSAIDs only)	2,550	14.5	[12.0−17.7]
- Potassium-sparing diuretics	No diuretics (RASIs + NSAIDs only)	426	2.86	[1.82−4.48]
- Thiazide diuretics	No diuretics (RASIs + NSAIDs only)	775	1.38	[0.95−1.98]
- Thiazide-like diuretics	No diuretics (RASIs + NSAIDs only)	239	2.00	[1.06−3.78]
- V2RA	No diuretics (RASIs + NSAIDs only)	17	NE	
Exactly 2 diuretics				
- Any combination	1 diuretic (any diuretics)	1,933	6.59	[5.33−8.15]
- Loop + Potassium-sparing	Loop only	856	5.66	[4.06−7.89]
- Loop + Thiazide	Loop only	232	2.34	[1.26−4.35]
- Loop + Thiazide-like	Loop only	57	6.59	[2.06−21.1]
- Loop + V2RA	Loop only	203	3.10	[1.57−6.11]

- Loop + Potassium-sparing	Potassium-sparing only	252	4.89	[2.54−9.43]
- Potassium-sparing + Thiazide	Potassium-sparing only	35	0.64	[0.15−2.70]
- Potassium-sparing + Thiazide-like	Potassium-sparing only	12	1.23	[0.10−14.5]
- Potassium-sparing + V2RA	Potassium-sparing only	6	NE	
- Loop + Thiazide	Thiazide only	355	23.7	[13.6−41.5]
- Potassium-sparing + Thiazide	Thiazide only	83	8.30	[2.08−33.1]
- Thiazide + Thiazide-like	Thiazide only	30	NE	
- Thiazide + V2RA	Thiazide only	5	NE	
- Loop + Thiazide-like	Thiazide-like only	78	161.0	[28.2−924]
- Potassium-sparing + Thiazide-like	Thiazide-like only	13	NE	
- Thiazide + Thiazide-like	Thiazide-like only	29	NE	
- Thiazide-like + V2RA	Thiazide-like only	2	NE	
- Loop + V2RA	V2RA only	13	3.75	[0.20−69.2]
- Potassium-sparing + V2RA	V2RA only	7	NE	
- Thiazide + V2RA	V2RA only	2	NE	
- Thiazide-like + V2RA	V2RA only	0	NE	
≥3 diuretics	V2RA only			
- Any combination	2 diuretics (Any combination)	487	3.66	[2.38−5.63]
Sensitivity analysis restricted to the period prior to the first AKI event				
Exactly 1 diuretic				
- Any diuretics	No diuretics (RASIs + NSAIDs only)	3,313	14.90	[12.4−17.9]
- Loop diuretics	No diuretics (RASIs + NSAIDs only)	2,295	44.1	[35.1−55.4]
- Potassium-sparing diuretics	No diuretics (RASIs + NSAIDs only)	351	3.83	[2.30−6.36]
- Thiazide diuretics	No diuretics (RASIs + NSAIDs only)	691	1.16	[0.79−1.71]
- Thiazide-like diuretics	No diuretics (RASIs + NSAIDs only)	222	1.80	[0.96−3.39]
- V2RA	No diuretics (RASIs + NSAIDs only)	11	NE	
Exactly 2 diuretics				
- Any combination	1 diuretic (any diuretics)	1,666	11.20	[8.82−14.3]

- Loop + Potassium-sparing	Loop only	724	7.10	[4.85−10.4]
- Loop + Thiazide	Loop only	165	2.75	[1.31−5.78]
- Loop + Thiazide-like	Loop only	37	1.99	[0.40−9.91]
- Loop + V2RA	Loop only	153	7.55	[3.37−16.9]
- Loop + Potassium-sparing	Potassium-sparing only	214	8.59	[4.16−17.8]
- Potassium-sparing + Thiazide	Potassium-sparing only	11	1.45	[0.10−21.1]
- Potassium-sparing + Thiazide-like	Potassium-sparing only	2	NE	
- Potassium-sparing + V2RA	Potassium-sparing only	9	NE	
- Loop + Thiazide	Thiazide only	324	41.5	[22.3−77.2]
- Potassium-sparing + Thiazide	Thiazide only	78	13.30	[2.65−66.2]
- Thiazide + Thiazide-like	Thiazide only	25	NE	
- Thiazide + V2RA	Thiazide only	5	NE	
- Loop + Thiazide-like	Thiazide-like only	69	243.0	[42.8−1382]
- Potassium-sparing + Thiazide-like	Thiazide-like only	16	NE	
- Thiazide + Thiazide-like	Thiazide-like only	28	NE	
- Thiazide-like + V2RA	Thiazide-like only	0	NE	
- Loop + V2RA	V2RA only	10	14.70	[0.71−301]
- Potassium-sparing + V2RA	V2RA only	3	NE	
- Thiazide + V2RA	V2RA only	1	NE	
- Thiazide-like + V2RA	V2RA only	0	NE	
≥3 diuretics	V2RA only			
- Any combination	2 diuretics (Any combination)	421	6.74	[4.15−10.9]

IRRs were estimated using a conditional Poisson regression in the SCCS sensitivity model, including time-dependent covariates (antibiotics, antiviral agents, and iodinated contrast media). Any diuretic indicated the use of exactly one diuretic agent from any class (loop, potassium-sparing, thiazide, thiazide-like, or V2RA). Any combination indicated the use of two or three diuretics from any class. The reference refers to the designated control exposure period for each comparison. NE (not estimable) indicates that the IRR could not be calculated because of insufficient AKI events.

Abbreviations: AKI, acute kidney injury. CI, confidence interval. IRR, incidence rate ratio. NSAIDs, nonsteroidal anti-inflammatory drugs. RASIs, renin–angiotensin system inhibitors. V2RA, vasopressin V2 receptor antagonist

Supplementary Table S5 Incidence rate ratios for AKI according to the type of concomitant RASIs under diuretic and NSAID exposure in sensitivity analyses

Exposure category	Reference	Patients, n	IRR	[95% CI]
Sensitivity analysis adjusting for exposure to antibiotics, antivirals, and iodinated contrast media				
ARB + diuretics + NSAIDs	ACEI + diuretics + NSAIDs	308	1.52	[0.91−2.56]
ARB + exactly 1 diuretic + NSAIDs	ACEI + exactly 1 diuretic + NSAIDs	160	1.05	[0.46−2.41]
Sensitivity analysis adjusting for antibiotics, antivirals, iodinated contrast media, and hospitalization periods				
ARB + diuretics + NSAIDs	ACEI + diuretics + NSAIDs	308	1.53	[0.91−2.58]
ARB + exactly 1 diuretic + NSAIDs	ACEI + exactly 1 diuretic + NSAIDs	160	1.07	[0.46−2.46]
Sensitivity analysis stratified by age (<75 years)				
ARB + diuretics + NSAIDs	ACEI + diuretics + NSAIDs	28	1.00	[0.20−4.90]
ARB + exactly 1 diuretic + NSAIDs	ACEI + exactly 1 diuretic + NSAIDs	10	0.41	[0.03−5.68]
Sensitivity analysis stratified by age (≥75 years)				
ARB + diuretics + NSAIDs	ACEI + diuretics + NSAIDs	280	1.56	[0.88−2.76]
ARB + exactly 1 diuretic + NSAIDs	ACEI + exactly 1 diuretic + NSAIDs	150	1.39	[0.54−3.57]
Sensitivity analysis restricted to the period prior to the first AKI event				
ARB + diuretics + NSAIDs	ACEI + diuretics + NSAIDs	183	1.49	[0.75−2.96]
ARB + exactly 1 diuretic + NSAIDs	ACEI + exactly 1 diuretic + NSAIDs	91	2.01	[0.55−7.31]

IRRs were estimated using a conditional Poisson regression in the SCCS sensitivity model, including time-dependent covariates (antibiotics, antiviral agents, and iodinated contrast media). The reference refers to the designated control exposure period for each comparison.

Abbreviations: ACEI, angiotensin-converting enzyme inhibitor. AKI, acute kidney injury. ARB, angiotensin II receptor blocker. CI, confidence interval. IRR, incidence rate ratio. NSAIDs, nonsteroidal anti-inflammatory drugs. RASIs, renin–angiotensin system inhibitors.

Supplementary Table S6 Incidence rate ratios for AKI according to the type of concomitant NSAIDs under RASI and diuretic exposure in the sensitivity analyses

Exposure category	Reference	Patients, n	IRR	[95% CI]
Sensitivity analysis adjusting for exposure to antibiotics, antivirals, and iodinated contrast media				
RASIs + diuretics + nsCOXi	RASIs + diuretics + sCOX-2i	2,277	0.85	[0.69−1.04]
RASIs + exactly 1 diuretic + nsCOXi	RASIs + exactly 1 diuretic + sCOX-2i	1,616	0.96	[0.73−1.26]
Sensitivity analysis adjusting for antibiotics, antivirals, iodinated contrast media, and hospitalization periods				
RASIs + diuretics + nsCOXi	RASIs + diuretics + sCOX-2i	2,277	0.82	[0.67−1.01]
RASIs + exactly 1 diuretic + nsCOXi	RASIs + exactly 1 diuretic + sCOX-2i	1,616	0.94	[0.71−1.23]
Sensitivity analysis stratified by age (<75 years)				
RASIs + diuretics + nsCOXi	RASIs + diuretics + sCOX-2i	309	1.14	[0.66−1.99]
RASIs + exactly 1 diuretic + nsCOXi	RASIs + exactly 1 diuretic + sCOX-2i	232	1.19	[0.59−2.39]
Sensitivity analysis stratified by age (≥75 years)				
RASIs + diuretics + nsCOXi	RASIs + diuretics + sCOX-2i	1,968	1.38	[1.10−1.74]
RASIs + exactly 1 diuretic + nsCOXi	RASIs + exactly 1 diuretic + sCOX-2i	1,384	1.64	[1.22−2.22]
Sensitivity analysis restricted to the period prior to the first AKI event				
RASIs + diuretics + nsCOXi	RASIs + diuretics + sCOX-2i	1,686	1.25	[0.97−1.62]
RASIs + exactly 1 diuretic + nsCOXi	RASIs + exactly 1 diuretic + sCOX-2i	1,215	1.41	[1.01−1.96]

IRRs were estimated using a conditional Poisson regression in the SCCS sensitivity model, including time-dependent covariates (antibiotics, antiviral agents, and iodinated contrast media). The reference refers to the designated control exposure period for each comparison.

Abbreviations: AKI, acute kidney injury. CI, confidence interval. IRR, incidence rate ratio. NSAIDs, nonsteroidal anti-inflammatory drugs. nsCOXi, nonselective cyclooxygenase inhibitors. RASIs, renin–angiotensin system inhibitors. sCOX-2i, selective cyclooxygenase-2 inhibitors.
